# Supplementary material for: Molecular insights into the dual activation of PomZ, a ParA/MinD P-loop ATPase, by the two ATPase-activating proteins PomX and PomY
Source: mBio. 2026 Apr 15;17(5):e00425-26. doi: 10.1128/mbio.00425-26 (PMC13170346; doi:10.1128/mbio.00425-26)
Supplement: Supplemental Information — Figures S1-S14; Tables S1-S4. [file mbio.00425-26-s0001.pdf]

Supplementary Information

Molecular insights into the dual activation of PomZ, a ParA/MinD P-loop ATPase, by the two ATPase activating proteins PomX and PomY

Philipp Klos, Andrea Harms & Lotte Søgaard-Andersen

This file contains:
- Supplementary Figures 1-14
- Supplementary Tables 1-4
- Supplementary References

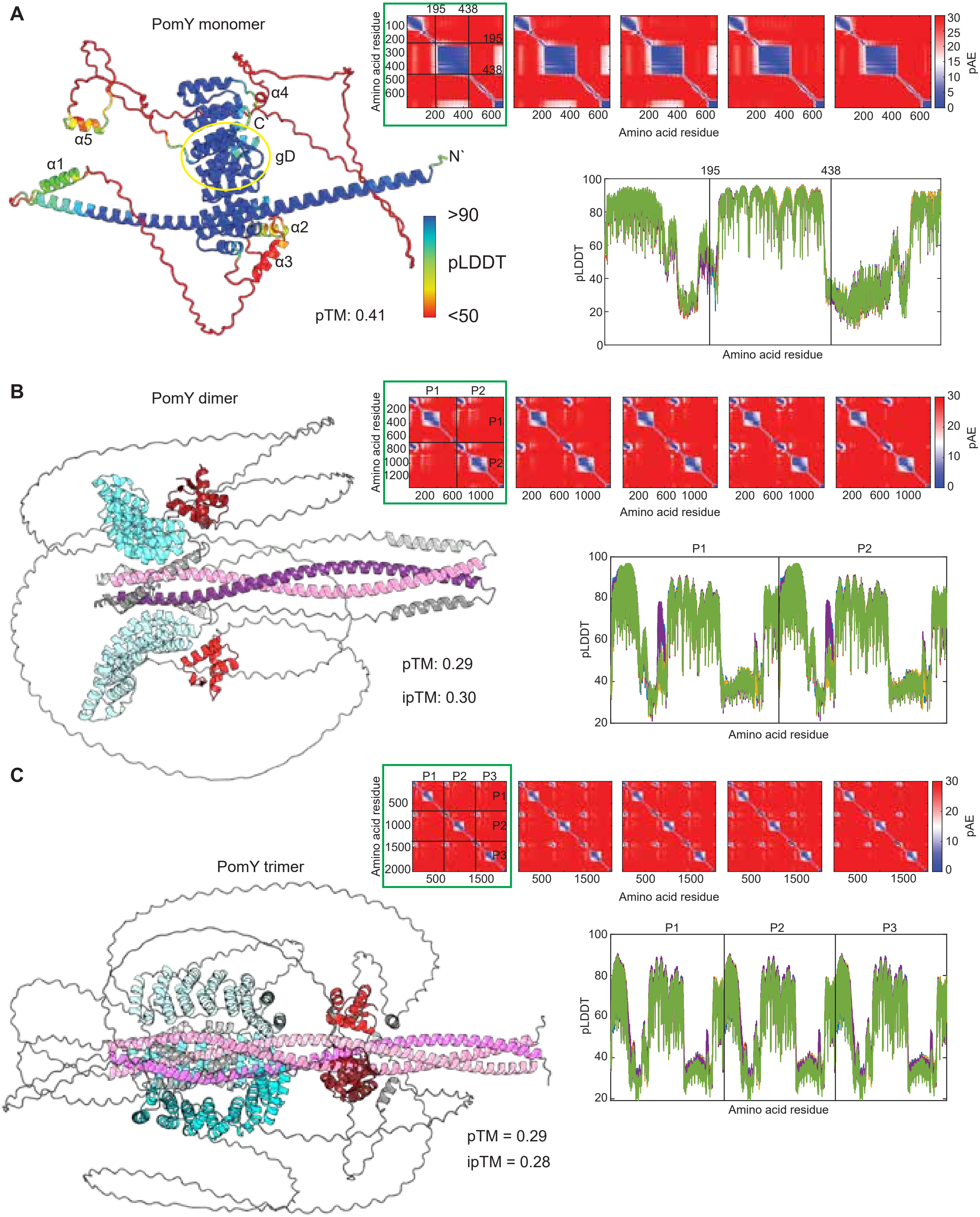
A
PomY monomer
α1
α2
α3
α4
α5
C`
N`
gD
>90
pLDDT
<50
pTM: 0.41
195
438
Amino acid residue
pAE
pLDDT
B
PomY dimer
P1
P2
pTM: 0.29
ipTM: 0.30
Amino acid residue
pAE
pLDDT
C
PomY trimer
P1
P2
P3
pTM = 0.29
ipTM = 0.28
Amino acid residue
pAE
pLDDT

Figure S1. AlphaFold3-based structural models of PomY.

(A) Left panel, model rank 1 of PomY monomer colored according to pLDDT score. The gD domain is indicated by a yellow circle. Right panels, pAE and pLDDT plots of the five models generated. Black lines indicate truncation points described in Fig. 1A and Fig. 2B. Model rank 1 (highlighted by a green box) was selected for further analyses.

(B-C) Left panel, model rank 1 of PomY dimer (B) and trimer (C). Domains are colored as in Fig. 1A. Right panels in, pAE and pLDDT plots of the five models generated. Black lines separate the two (B) and three (C) protomers. Model rank 1 (highlighted by a green box) was selected for further analysis. Note that the pTM and ipTM scores for both the dimer and trimer are below 0.50 (Fig. S1B-C). Because the pTM and ipTM scores reflect the confidence of the entire structure, the overall confidence of these structures is low due to the large proportion IDRs.

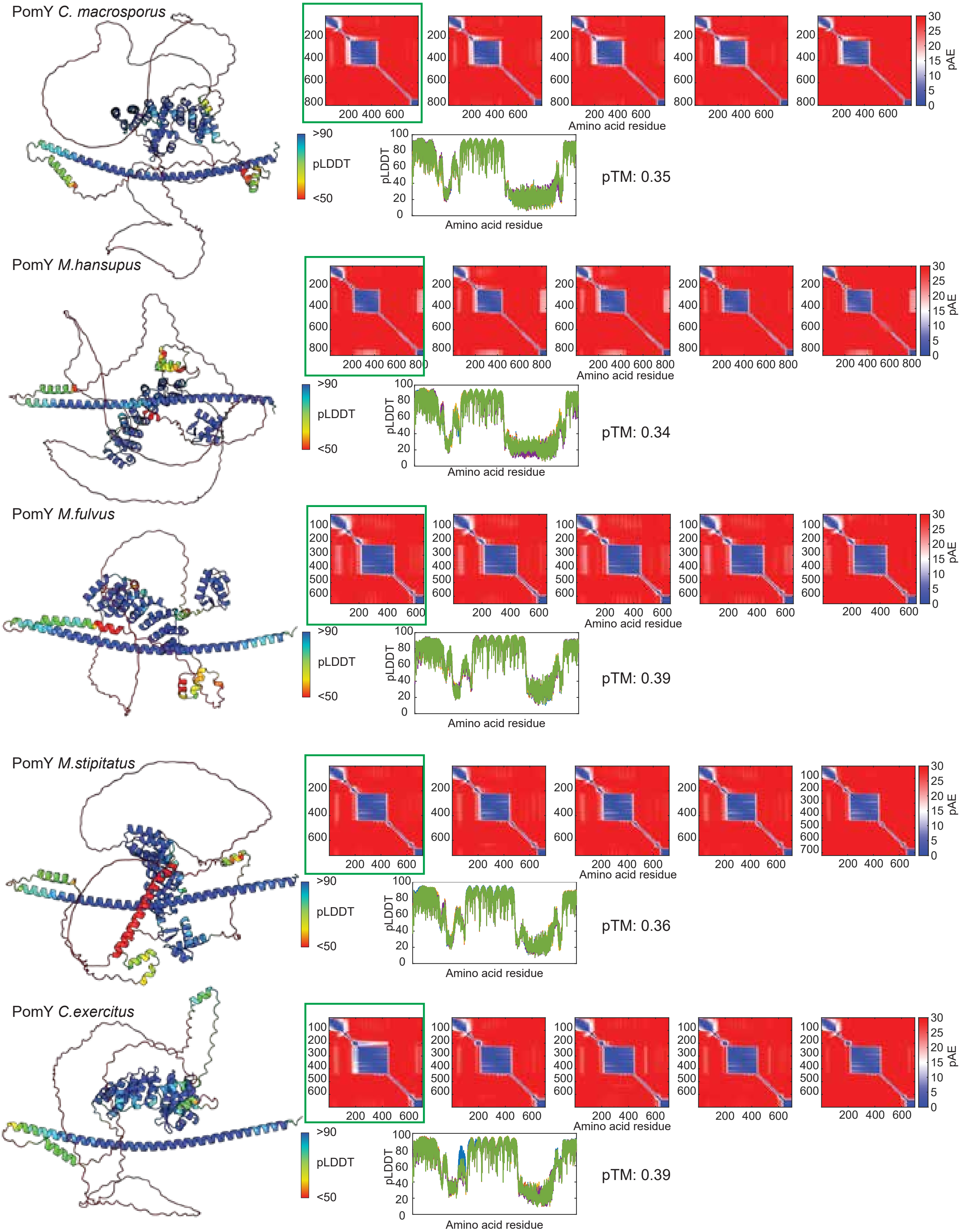
PomY C. macrosporus
pLDDT
>90
<50
Amino acid residue
pAE
pTM: 0.35
PomY M.hansupus
pTM: 0.34
PomY M.fulvus
pTM: 0.39
PomY M.stipitatus
pTM: 0.36
PomY C.exercitus
pTM: 0.39

Figure S2. AlphaFold3-based structural models of monomeric PomY orthologs.
Left panels, model rank 1 of the indicated PomY ortholog colored according to pLDDT scores. Right panels, pAE and pLDDT plots of the five models generated. Model rank 1 (highlighted by a green box) was selected for further analysis. Note that the models are of low confidence based on the pTM scores. The large proportion of IDRs, which have low pLDDT scores, causes these low scores.

PomY *C.interemptor*

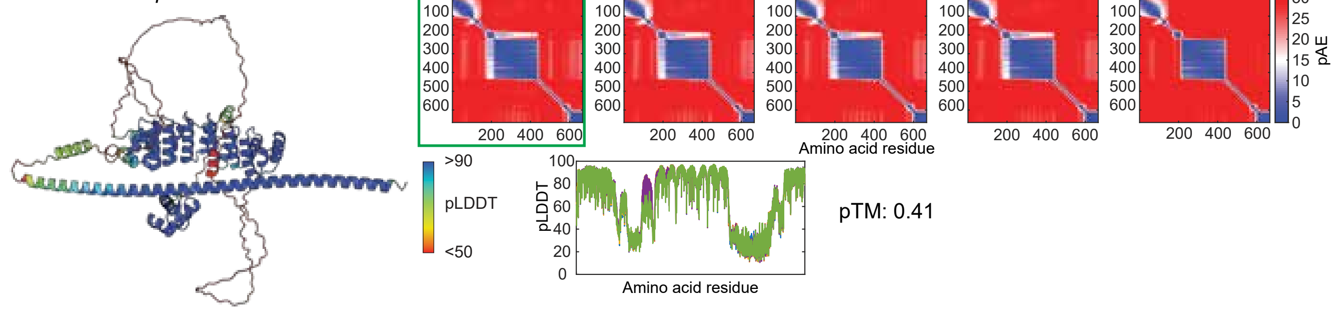

PomY *C.coralloides*

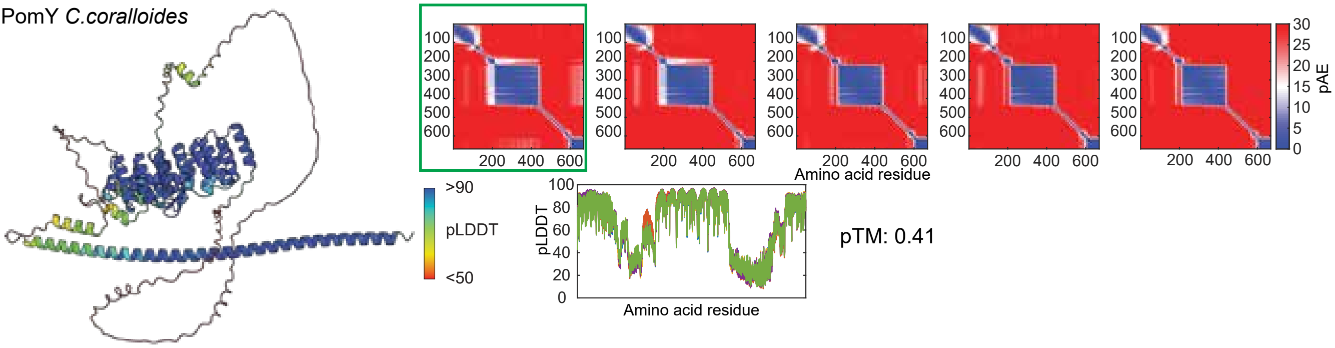

PomY *M.boletus*

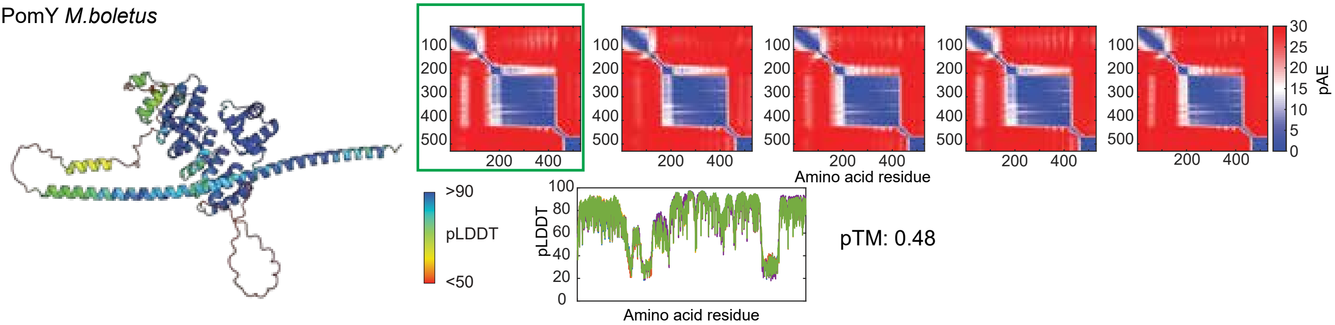

PomY *C.fuscus*

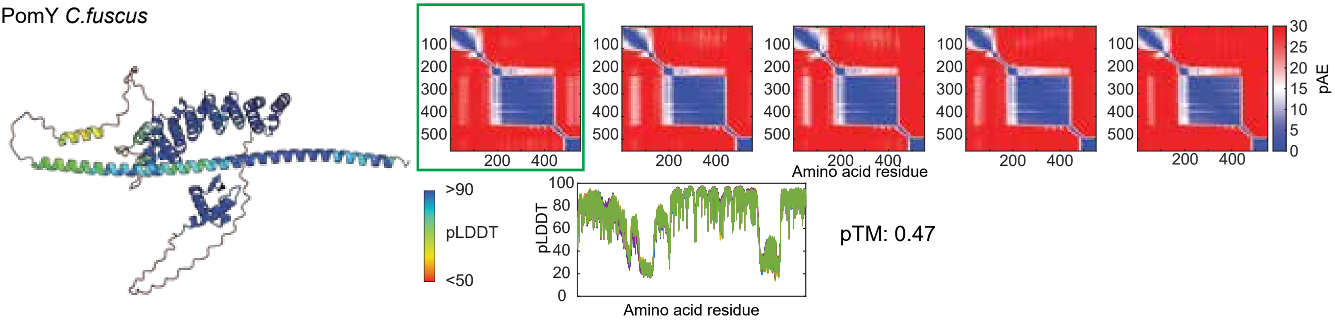

PomY *A.gephyra*

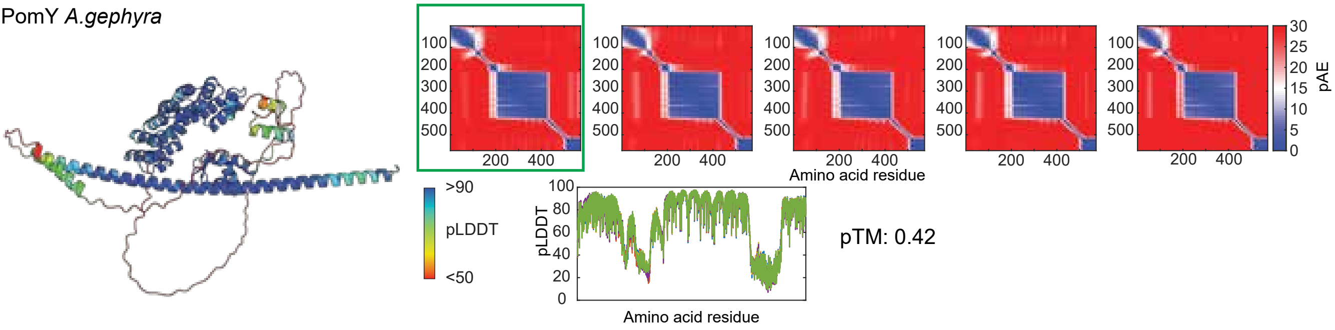

Figure S3. AlphaFold3-based structural models of monomeric PomY orthologs.
Left panels, model rank 1 of the indicated PomY ortholog colored according to pLDDT scores. Right panels, pAE and pLDDT plots of the five models generated. Model rank 1 (highlighted by a green box) was selected for further analysis. Note that the models are of low confidence based on the pTM scores. The large proportion of IDRs, which have low pLDDT scores, causes these low scores.

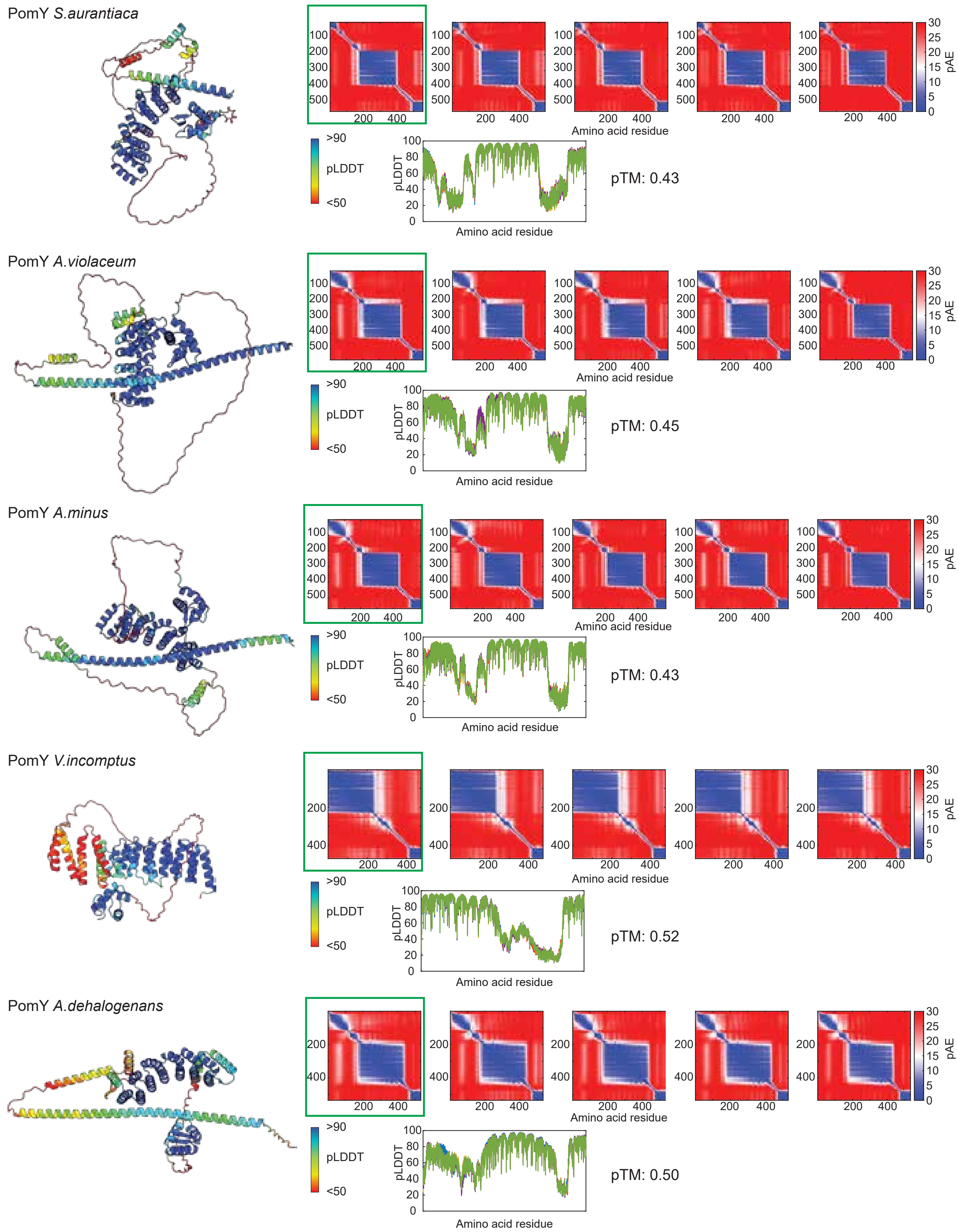

PomY S.aurantiaca
100
200
300
400
500
200
400
Amino acid residue
30
25
20
15
10
5
0
pAE
>90
pLDDT
<50
100
80
60
40
20
0
pLDDT
Amino acid residue
pTM: 0.43
PomY A.violaceum
pTM: 0.45
PomY A.minus
pTM: 0.43
PomY V.incomptus
pTM: 0.52
PomY A.dehalogenans
pTM: 0.50

Figure S4. AlphaFold3-based structural models of monomeric PomY orthologs.
Left panels, model rank 1 of the indicated PomY ortholog colored according to pLDDT scores. Right panels, pAE and pLDDT plots of the five models generated. Model rank 1 (highlighted by a green box) was selected for further analysis. Note that the models are of low confidence based on the pTM scores. The large proportion of IDRs, which have low pLDDT scores, causes these low scores.

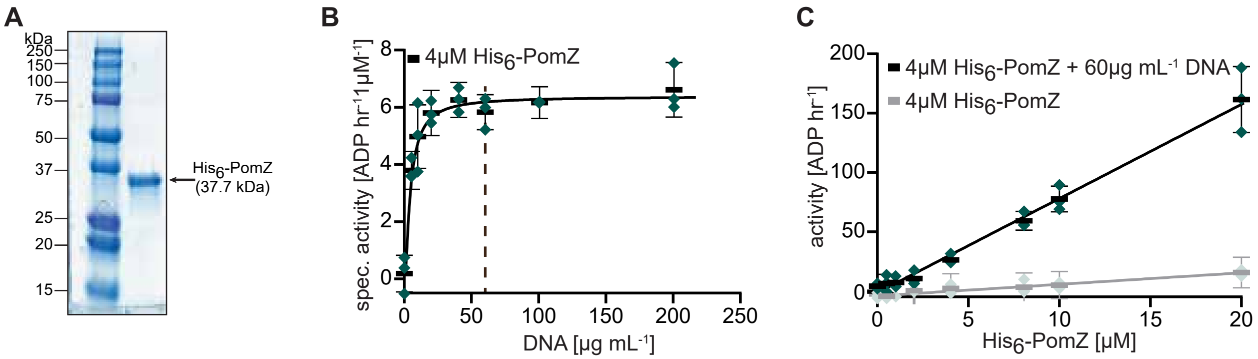

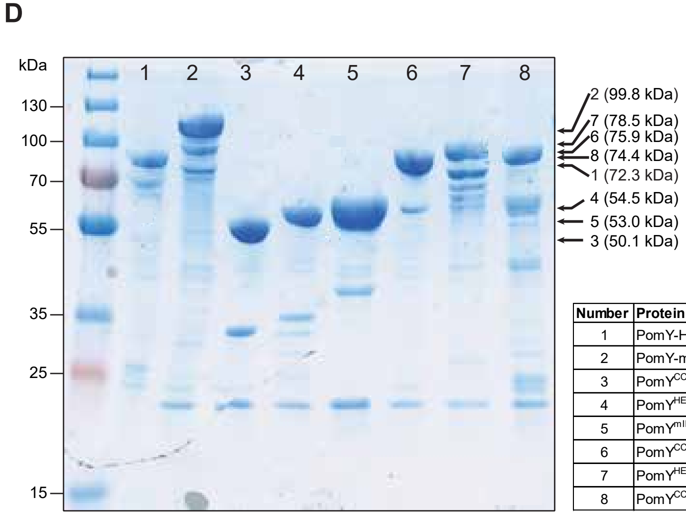

Number	Protein	Mass [kDa]
1	PomY-His$_6$	72.3
2	PomY-mCh-His$_6$	99.8
3	PomY^{CC}-mCh-His$_6$	50.1
4	PomY^{HEAT}-mCh-His$_6$	54.5
5	PomY^{mIDR}-mCh-His$_6$	53.0
6	PomY^{CC_HEAT}-mCh-His$_6$	75.9
7	PomY^{HEAT_mIDR}-mCh-His$_6$	78.5
8	PomY^{CC_mIDR}-mCh-His$_6$	74.4

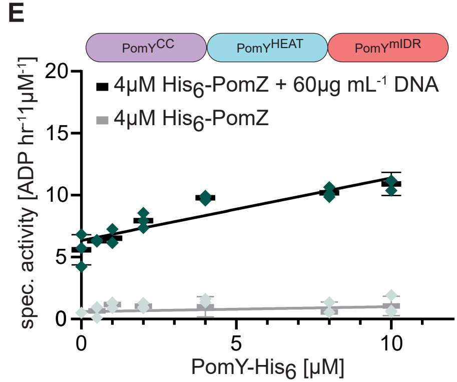

Figure S5. Purification and analysis of PomZ and PomY variants.
(A) SDS-PAGE analysis of purified His_6-PomZ. Molecular size markers are shown on the left and purified His_6-PomZ including calculated MW on the right.
(B) His_6-PomZ ATPase activity. Double stranded herring sperm DNA (DNA) was added as indicated. The assay was performed as described in Fig. 2A. Green points represent one biological replicate, which was calculated as the mean of three technical replicates. Black bars and error bars, mean of three biological replicates and STDEV. Spontaneous ATP hydrolysis and NADH consumption were accounted for by subtracting the measurements in the absence of His_6-PomZ.
(C) His_6-PomZ ATPase activity. His_6-PomZ and DNA were added as indicated. The assay was performed and analysed as in (B).
(D) SDS-PAGE analysis of purified PomY variants. Left lane, molecular size markers with indicated masses. Each protein was assigned a number, which is detailed in the table on the right. As previously described, PomY-His_6 as well as PomY-mCh migrated aberrantly in SDS-PAGE (1, 2). Proteins were purified as mCh-His_6-tagged proteins; in the main text, they are referred to as mCh-tagged proteins for simplicity.
(E) His_6-PomZ ATPase activity. PomY-His_6 and DNA was added as indicated. The assay was performed and analysed as in (B).

A

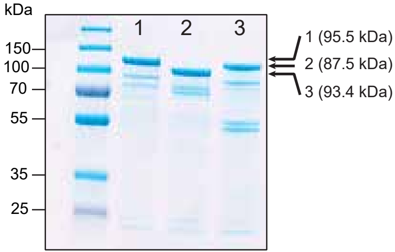

Number	Protein	Mass [kDa]
1	PomY$^{\Delta 439\text{-}488}$-mCh-His$_6$	95.5
2	PomY$^{\Delta 496\text{-}623}$-mCh-His$_6$	87.5
3	PomY$^{\Delta 624\text{-}682}$-mCh-His$_6$	93.4

B

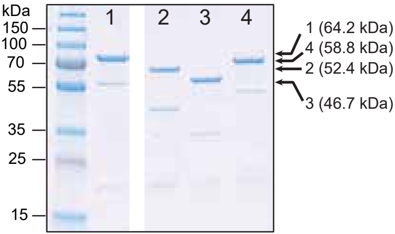

Number	Protein	Mass [kDa]
1	His$_6$-Sumo-PomY^{mIDR}-mCh	64.2
2	His$_6$-Sumo-PomY$^{496\text{-}623}$-mCh	52.4
3	His$_6$-Sumo-PomY$^{624\text{-}682}$-mCh	46.7
4	His$_6$-Sumo-PomY$^{496\text{-}682}$-mCh	58.8

C

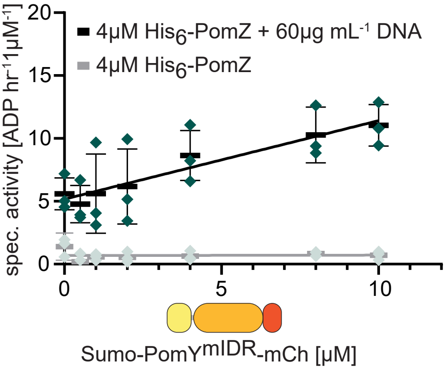

Figure S6. Purification of PomY variants.

(A-B) SDS-PAGE analysis of purified PomY variants. Left lane, molecular size markers with indicated masses. Each protein was assigned a number, which is detailed in the tables below. The proteins in (A) were purified as mCh-His_6-tagged proteins; in the main text, they are referred to as mCh-tagged proteins for simplicity. The proteins in (B) were purified as His_6-Sumo-tagged proteins; in the main text, they are referred to as Sumo-tagged proteins for simplicity. In B, proteins were separated on the same gel; the gap indicates lanes removed for presentation purposes.

(C) His_6-PomZ ATPase activity. Sumo-PomY^{mIDR}-mCh and DNA was added as indicated. The assay was performed and analysed as in Fig. 2A.

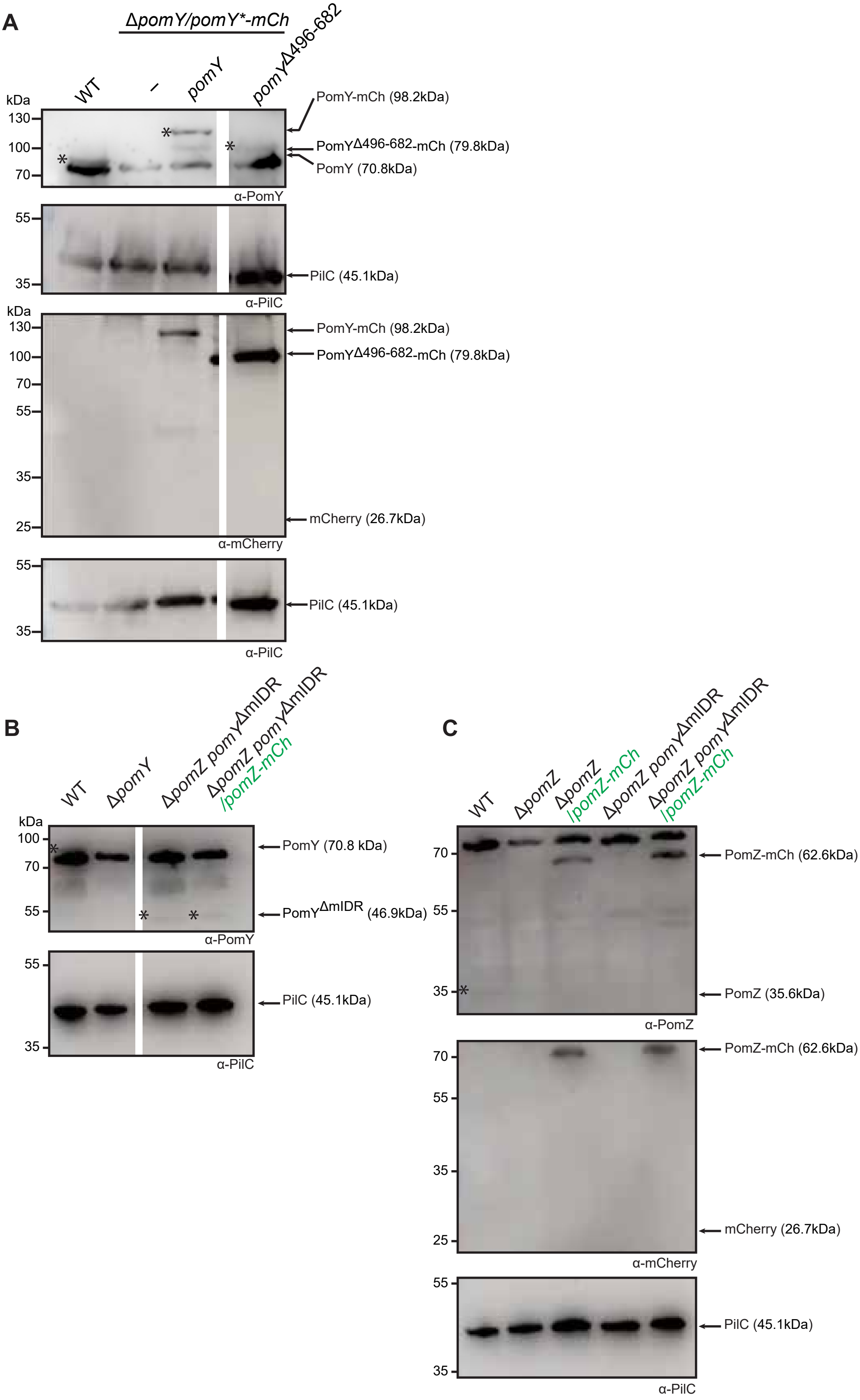
A
ΔpomY/pomY*-mCh
WT
–
pomY
pomYΔ496-682
kDa
130
100
70
PomY-mCh (98.2kDa)
PomYΔ496-682-mCh (79.8kDa)
PomY (70.8kDa)
α-PomY
55
35
PilC (45.1kDa)
α-PilC
kDa
130
100
70
55
35
25
PomY-mCh (98.2kDa)
PomYΔ496-682-mCh (79.8kDa)
mCherry (26.7kDa)
α-mCherry
55
35
PilC (45.1kDa)
α-PilC
B
WT
ΔpomY
ΔpomZ pomYΔmIDR
ΔpomZ pomYΔmIDR /pomZ-mCh
kDa
100
70
55
PomY (70.8 kDa)
PomYΔmIDR (46.9kDa)
α-PomY
55
35
PilC (45.1kDa)
α-PilC
C
WT
ΔpomZ
ΔpomZ /pomZ-mCh
ΔpomZ pomYΔmIDR
ΔpomZ pomYΔmIDR /pomZ-mCh
70
55
35
PomZ-mCh (62.6kDa)
PomZ (35.6kDa)
α-PomZ
70
55
35
25
PomZ-mCh (62.6kDa)
mCherry (26.7kDa)
α-mCherry
55
35
PilC (45.1kDa)
α-PilC

Figure S7. Immunoblot analysis of the accumulation level of PomY variants and PomZ-mCh in *M. xanthus*.

(A) Immunoblot analysis of PomY variants. Total cellular lysates from an equal amount of cells of the indicated *M. xanthus* strains were separated *via* SDS-PAGE, followed by immunoblotting with the indicated antibodies. PilC was used as a loading control. All samples were analysed on the same blot and the gap indicates lanes that were removed for presentation purposes. *, indicates (from left to right) PomY, PomY-mCh and PomY$^{\Delta 496\text{-}682}$-mCh.

(B) Immunoblot analysis of PomY variants. The analysis was done and presented as in (A). *, indicates (from left to right) PomY, PomY$^{\Delta mIDR}$ and PomY$^{\Delta mIDR}$.

(C) Immunoblot analysis of PomZ-mCh. The analysis was done and presented as in (A). *, indicates PomZ.

A

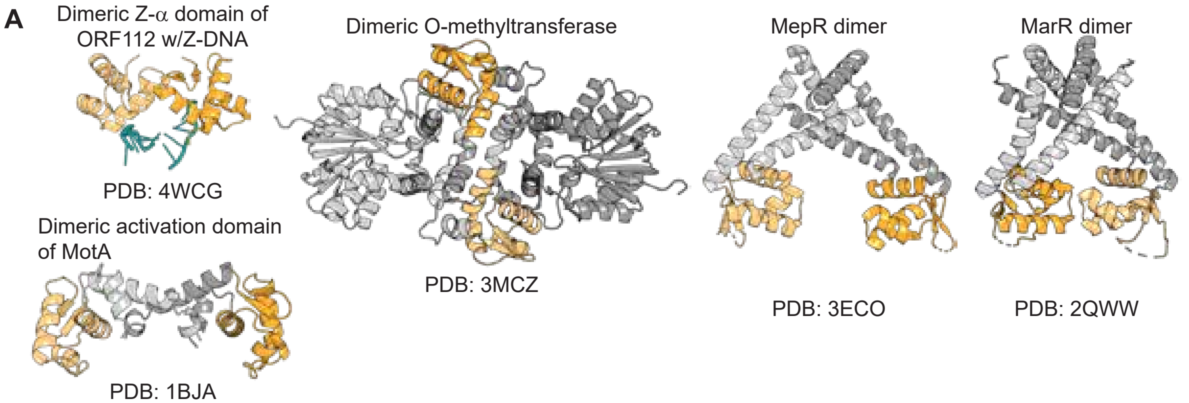

B

Protein	Description	Probability	Seq. Identiy	E-value	homologous region in gD domain
ORF112	wHTH domain in Z-DNA binding Z-α domain	1	20.3	8.58E-03	624 626 682
	wHTH domain of unknown function in O-methyltransferase	1	18.9	2.51E-03	624 627 682
MepR	wHTH domain in DNA binding domain of transcriptional regulator	1	14.5	2.23E-02	624 629 682
MarR	wHTH domain in DNA binding domain of transcriptional regulator	1	15.7	1.95E-02	624 628 682
MotA	wHTH domain in activation domain of T4 transcriptional regulator	1	12.7	1.21E-02	624 629 682

C

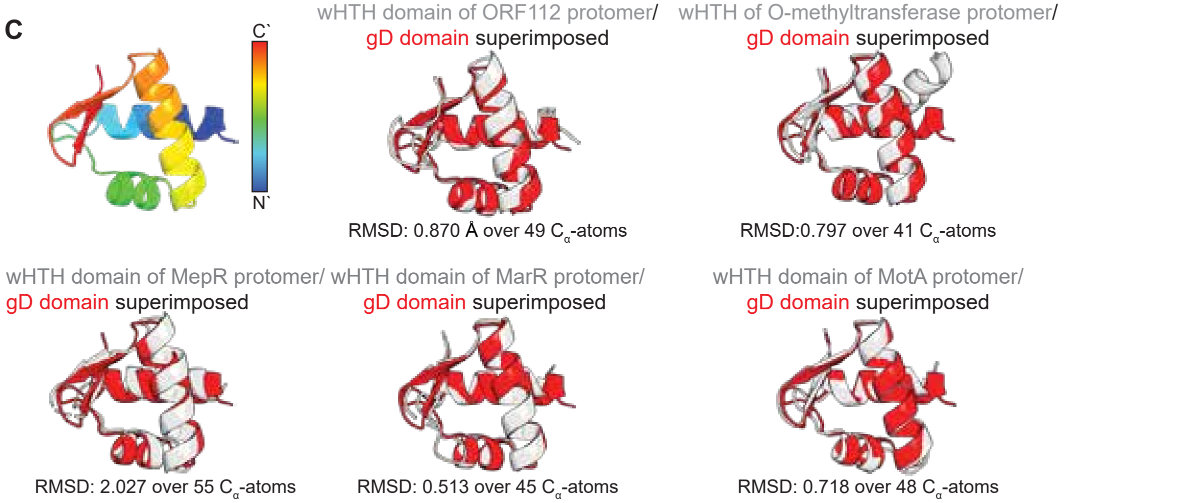

D

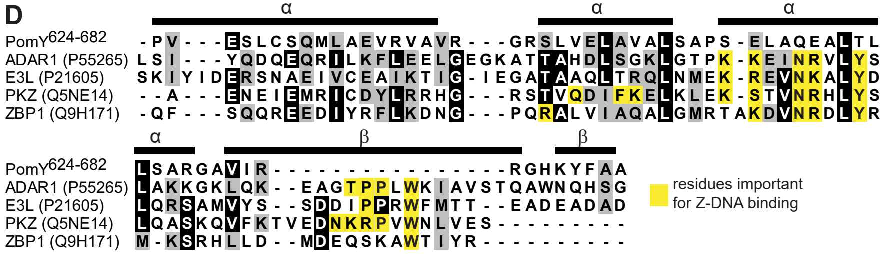

E

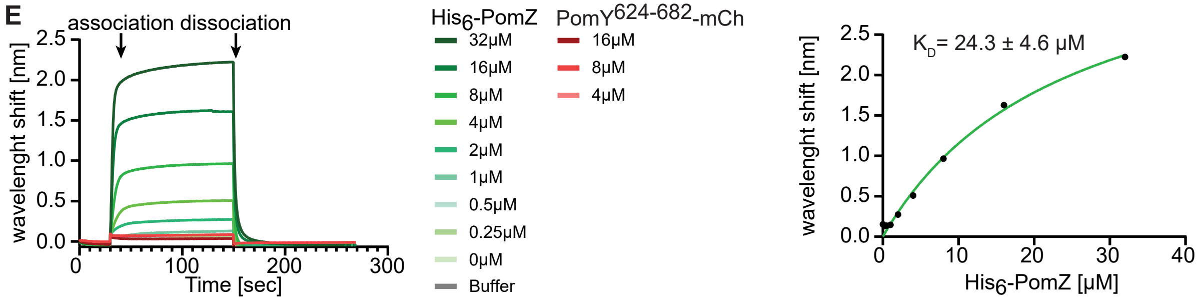

Figure S8. Computational and experimental analysis of the gD domain of PomY.
(A) Solved structures of the five best hits of the Foldseek analysis for structural homologs of the gD domain. The protein regions structurally aligning with the gD domain are marked in orange.
(B) Foldseek analysis of the gD domain against solved structures in the PDB database. From left to right: Protein name of structural homolog, description of the wHTH domain in the structural homologs, probability, sequence identity for structurally aligned residues, E-value; region of the gD domain aligning with the structural homolog.
(C) Structural alignment of the gD domain with the wHTH domains of the five best hits of the FoldSeek analysis. Upper left, the gD domain colored from the N- to the C-terminus; remaining five panels, structural alignment of the gD domain with the wHTH domains n which the gD domain is in red and the structural homologs in light grey.
(D) Multiple sequence alignment of the gD domain with Z-α domains. Secondary structure elements of the gD domain are indicated as black lines above the alignment. Residues involved in binding Z-DNA are marked in yellow (3-6).
(E) BLI analysis of the interaction of the gD domain and His_6-PomZ with double stranded DNA. Left panel, a Streptavidin-coated sensor was loaded with a double-biotinylated 226bp DNA fragment and probed with the indicated proteins at the indicated concentrations in the presence of 1mM ATP (association). The protein bound sensor was incubated in wash buffer to observe protein dissociation (dissociation). Note that $PomY^{624-682}$-mCh only contains the gD domain. Right panel, the wavelength shifts obtained for His_6-PomZ at the end of the association phase were plotted as a function of protein concentration and fitted to a one-site specific-binding model. The graph shows the mean of three biological replicates. The K_D value is the mean of the three biological replicates ±STDEV.

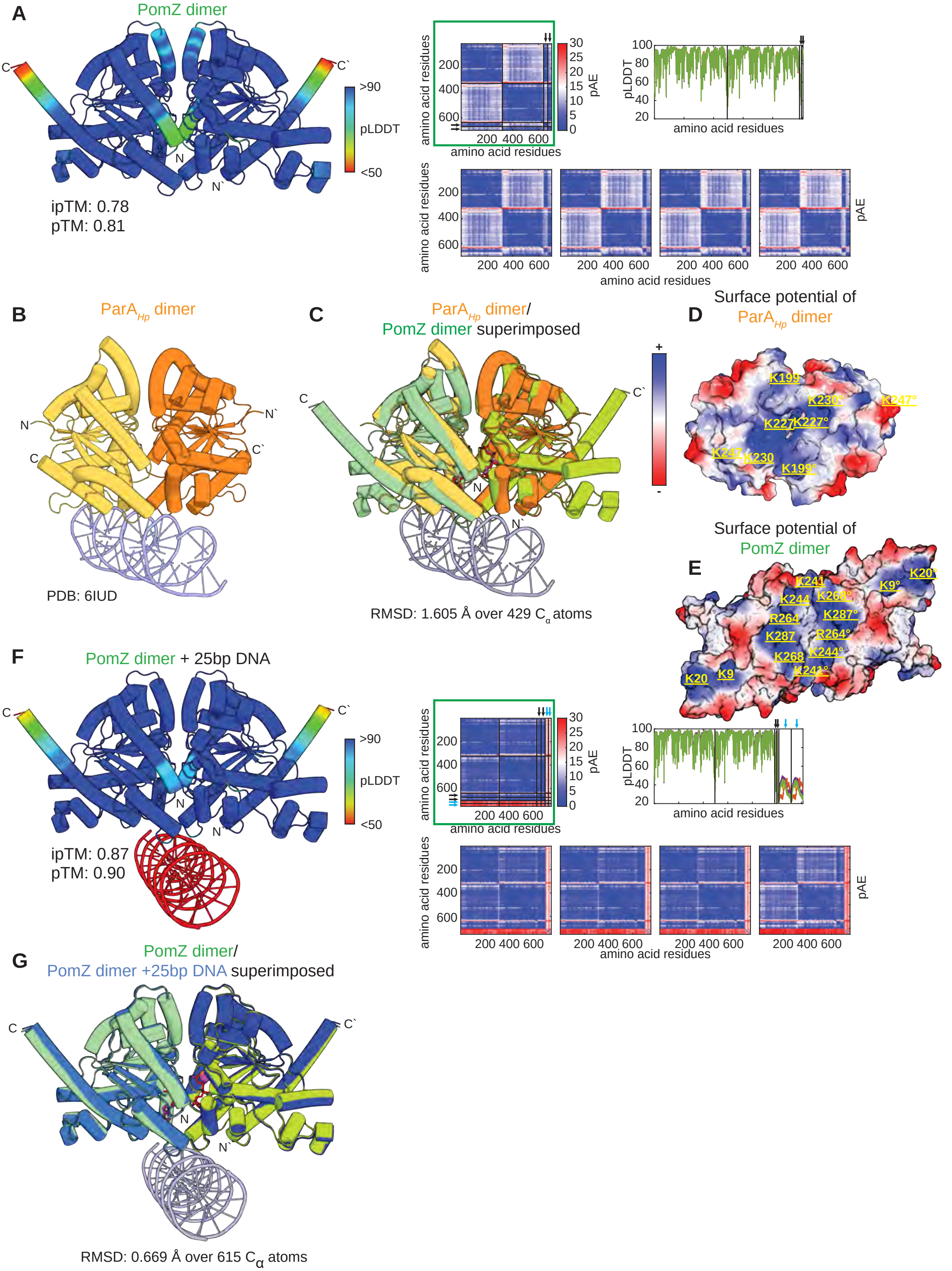
A
PomZ dimer
C
C`
N
N`
>90
pLDDT
<50
ipTM: 0.78
pTM: 0.81
amino acid residues
pAE
pLDDT
B
ParA$_{Hp}$ dimer
PDB: 6IUD
C
ParA$_{Hp}$ dimer/
PomZ dimer superimposed
RMSD: 1.605 Å over 429 C_α atoms
D
Surface potential of
ParA$_{Hp}$ dimer
K199
K230°
K247°
K227
K227°
K247
K230
K199°
E
Surface potential of
PomZ dimer
K241
K20°
K244
K268°
K9°
R264
K287°
K287
R264°
K268
K244°
K20
K9
K241°
F
PomZ dimer + 25bp DNA
ipTM: 0.87
pTM: 0.90
G
PomZ dimer/
PomZ dimer +25bp DNA superimposed
RMSD: 0.669 Å over 615 C_α atoms

Figure S9. AlphaFold3-based structural model of PomZ dimer.
(A) Left panel, model rank 1 of PomZ dimer colored according to pLDDT score; PomZ was modeled with ATP and Mg^{2+}. Right panels, pLDDT plot and pAE plots for the five generated models of the PomZ dimer. The black lines separate the two protomers; black arrows, ATP and Mg^{2+} molecules. Model rank 1 (highlighted by a green box) was selected for further analyses.
(B) Solved structure of ATP-bound *Helicobacter pylori* ParA dimer ($ParA_{Hp}$) bound to DNA (7).
(C) Structural alignment of PomZ dimer with $ParA_{Hp}$ bound to DNA. PomZ dimer is in shades of green, $ParA_{Hp}$ in shades of orange, and DNA in light blue. ATP molecules from PomZ model, red and Mg^{2+}, light orange. ADP molecules from $ParA_{HP}$ model, violet and Mg^{2+}, magenta.
(D-E) Electrostatic surface potential of $ParA_{Hp}$ dimer bound to DNA (C) and PomZ dimer (D) contoured from +5 to −5 kT e^{-1}. Residues marked in yellow are involved in DNA binding in the $ParA_{Hp}$ dimer and suggested to be involved in DNA binding in the PomZ dimer. Residues marked with ° distinguish between the two protomers.
(F) Left panel, model rank 1 of PomZ dimer bound to 25bp DNA fragment colored according to pLDDT values. PomZ was modeled with ATP and Mg^{2+} Right panel, pAE and pLDDT plots of the five models generated. The black lines separate the two protomers; black arrows, ATP and Mg^{2+} molecules, light blue arrows, DNA molecules.
(G) Structural alignment of PomZ dimer modelled in the absence of DNA and PomZ dimer modelled with DNA. PomZ dimer without DNA is in shades of green, PomZ dimer with DNA in blue. DNA is coloured in pale blue. ATP molecules from the PomZ model without DNA, red and Mg^{2+} ions, light orange; ATP molecules of the PomZ model with DNA, purple and Mg^{2+} ions, magenta.

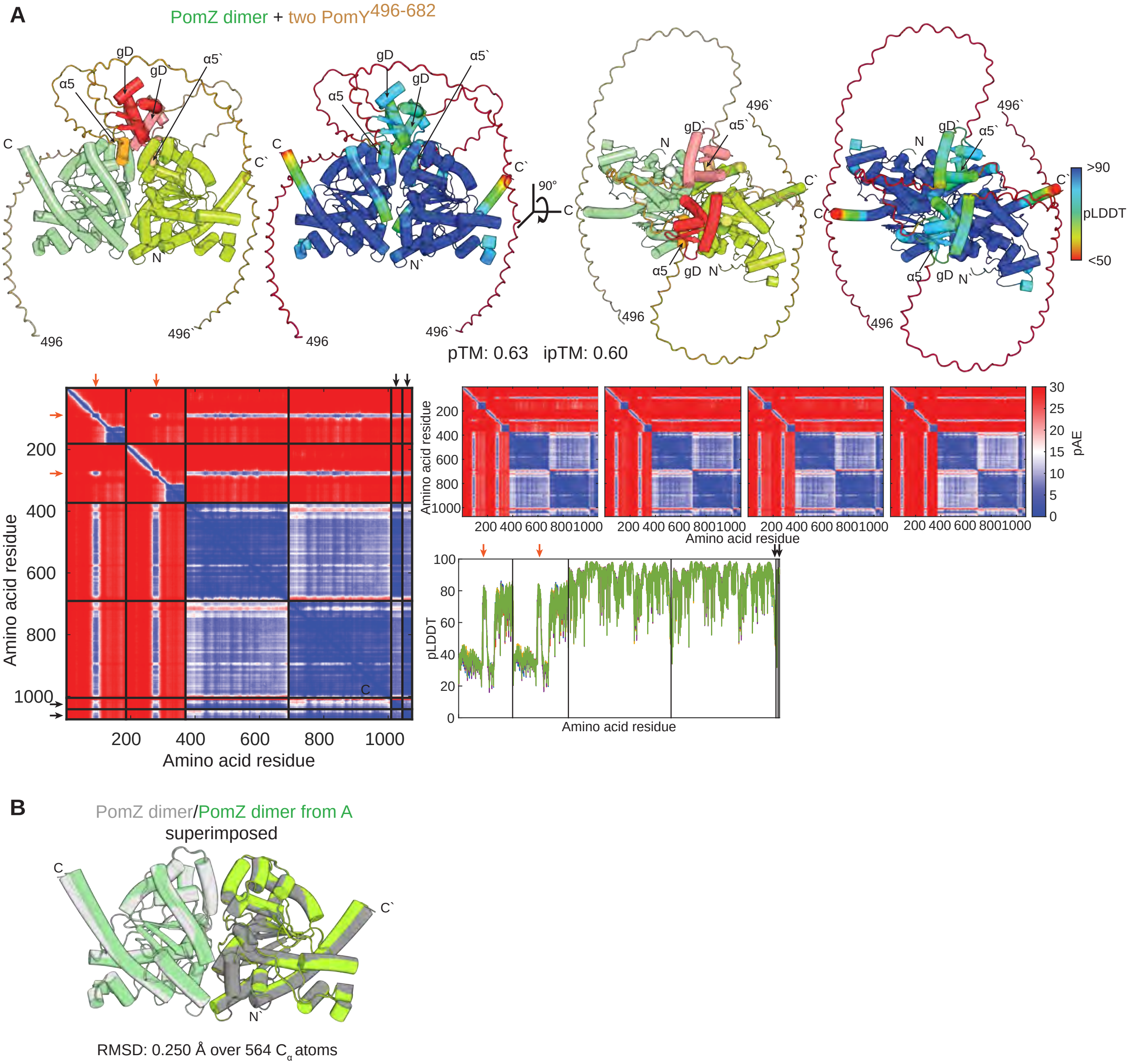
A
PomZ dimer + two PomY496-682
gD
gD`
α5
α5`
C
C`
N`
496
496`
90°
N
pLDDT
>90
<50
pTM: 0.63 ipTM: 0.60
Amino acid residue
pAE
pLDDT
B
PomZ dimer/PomZ dimer from A
superimposed
RMSD: 0.250 Å over 564 Cα atoms

Figure S10. AlphaFold3-based structural model of PomZ dimer in complex with PomY$^{496\text{-}682}$.
(A) Top panels, model rank 1 of PomZ dimer with two molecules of PomY$^{496\text{-}682}$. In the left panels, the model is shown with the PomZ dimer in shades of green, the IDRs in PomY$^{496\text{-}682}$ in brown, α5 in orange and the gD domain in red. In the right panels, the model is colored according to pLDDT score. PomZ was modeled with ATP and Mg^{2+} but the ATP molecules and Mg^{2+} are not included for simplicity. Lower panels, pLDDT plot and pAE plots for the five generated models. The black lines separate the two PomZ protomers and the two PomY$^{496\text{-}682}$ molecules; black arrows, ATP and Mg^{2+}, and orange arrows α5 of PomY$^{496\text{-}682}$. Model rank 1 is shown in high magnification and was selected for further analyses. Note that the model is of low confidence based on pTM and ipTM scores. The large proportion of IDRs in PomY$^{496\text{-}682}$, which have low pLDDT scores, causes these low values.
(B) Structural alignment of the PomZ dimer modelled in the absence of PomY$^{496\text{-}682}$ and in the presence of PomY$^{496\text{-}682}$. The PomZ dimer alone is in shades of grey and the PomZ dimer in the PomZ/PomY$^{496\text{-}682}$ complex is in shades of green.

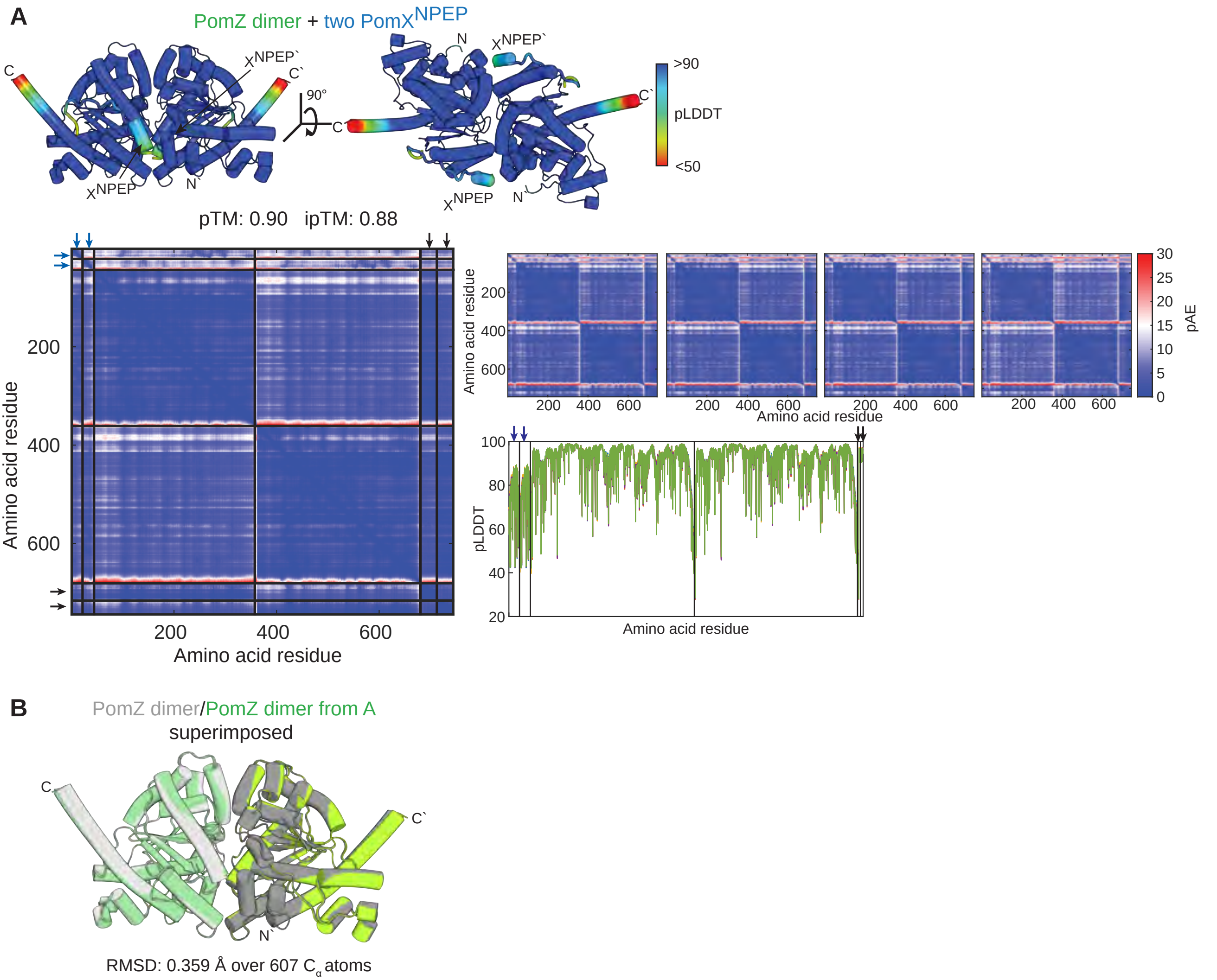
A
PomZ dimer + two PomX^NPEP
C
C`
X^NPEP`
X^NPEP
N`
90°
N
>90
pLDDT
<50
pTM: 0.90 ipTM: 0.88
Amino acid residue
200
400
600
pAE
30
25
20
15
10
5
0
pLDDT
100
80
60
40
20
B
PomZ dimer/PomZ dimer from A
superimposed
RMSD: 0.359 Å over 607 Cα atoms

Figure S11. AlphaFold3-based structural model of PomZ dimer in complex with $PomX^{NPEP}$.
(A) Top panels, model rank 1 of PomZ dimer with two molecules of $PomX^{NPEP}$ colored according to pLDDT score. PomZ was modeled with ATP and Mg^{2+} but the ATP molecules and Mg^{2+} are not included for simplicity. Lower panels, pLDDT plot and pAE plots for the five generated models. The black lines separate the two PomZ protomers and two $PomX^{NPEP}$ molecules; black arrows, ATP and Mg^{2+}, and blue arrows $PomX^{NPEP}$. Model rank 1 is shown in high magnification and was selected for further analyses.
(B) Structural alignment of the PomZ dimer modelled in the absence of $PomX^{NPEP}$ and in the presence of $PomX^{NPEP}$. The PomZ dimer alone is in shades of grey and the PomZ dimer in the PomZ/$PomX^{NPEP}$ complex is in shades of green.

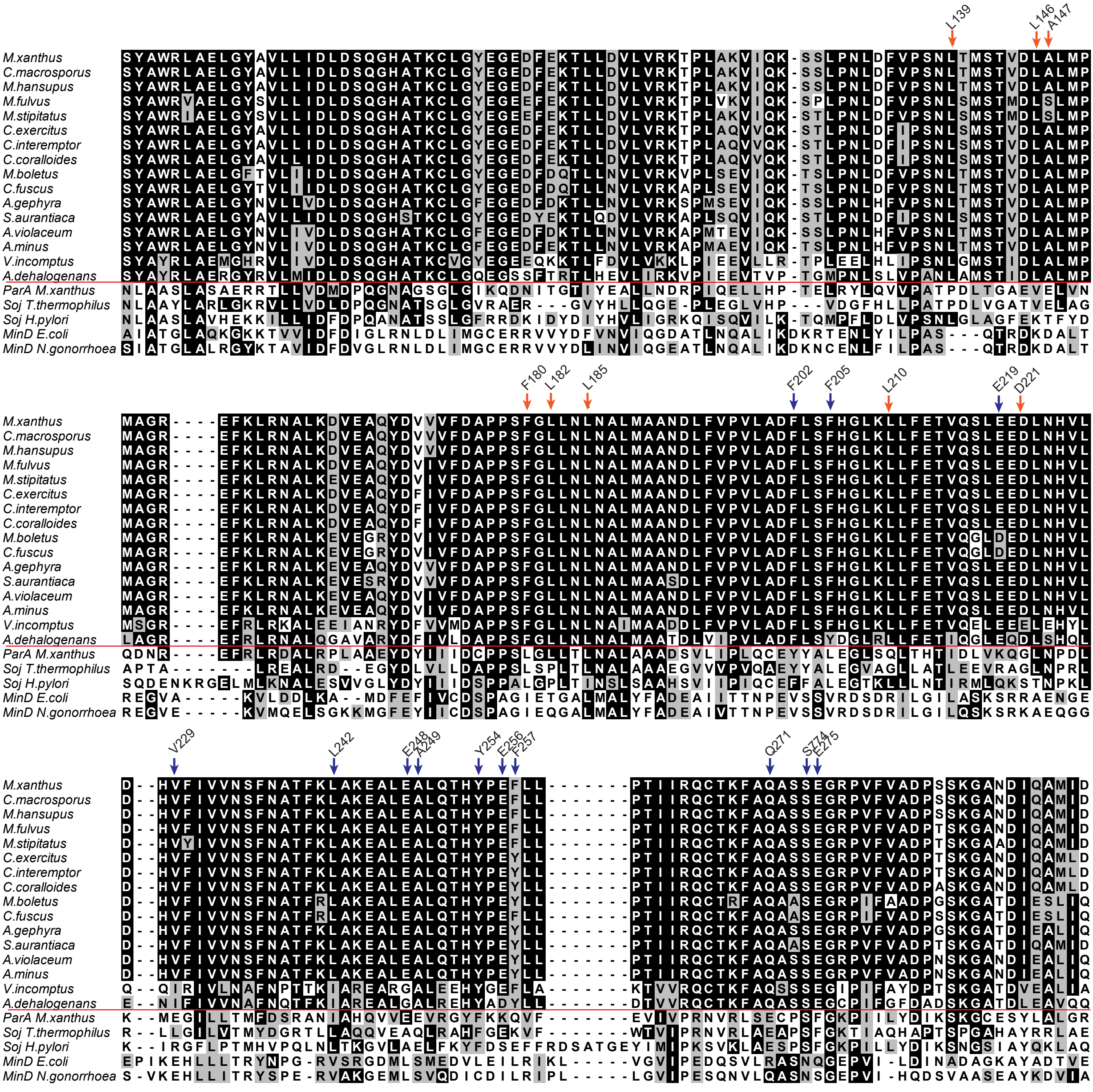
L139
L146
A147
M.xanthus
C.macrosporus
M.hansupus
M.fulvus
M.stipitatus
C.exercitus
C.interemptor
C.coralloides
M.boletus
C.fuscus
A.gephyra
S.aurantiaca
A.violaceum
A.minus
V.incomptus
A.dehalogenans
ParA M.xanthus
Soj T.thermophilus
Soj H.pylori
MinD E.coli
MinD N.gonorrhoea
F180
L182
L185
F202
F205
L210
E219
D221
V229
L242
E248
A249
Y254
E256
F257
Q271
S274
E275

Figure S12. Multiple sequence alignment of PomZ orthologs from Myxobacteria with fully sequenced genomes as well as ParA orthologs and MinD orthologs.
The alignment is visualized with a shading threshold of 80% based on the BLOSUM62 matrix. Grey boxes indicate similarity and black boxes identity. Orange arrows indicate residues in PomZ of *M. xanthus* predicted to interact with α5 of $PomY^{496\text{-}682}$. Blue arrows indicate residues predicted to interact with $PomX^{NPEP}$. Note that residue A147 is predicted to interact with α5 of $PomY^{496\text{-}682}$ only in the combined binding mode.

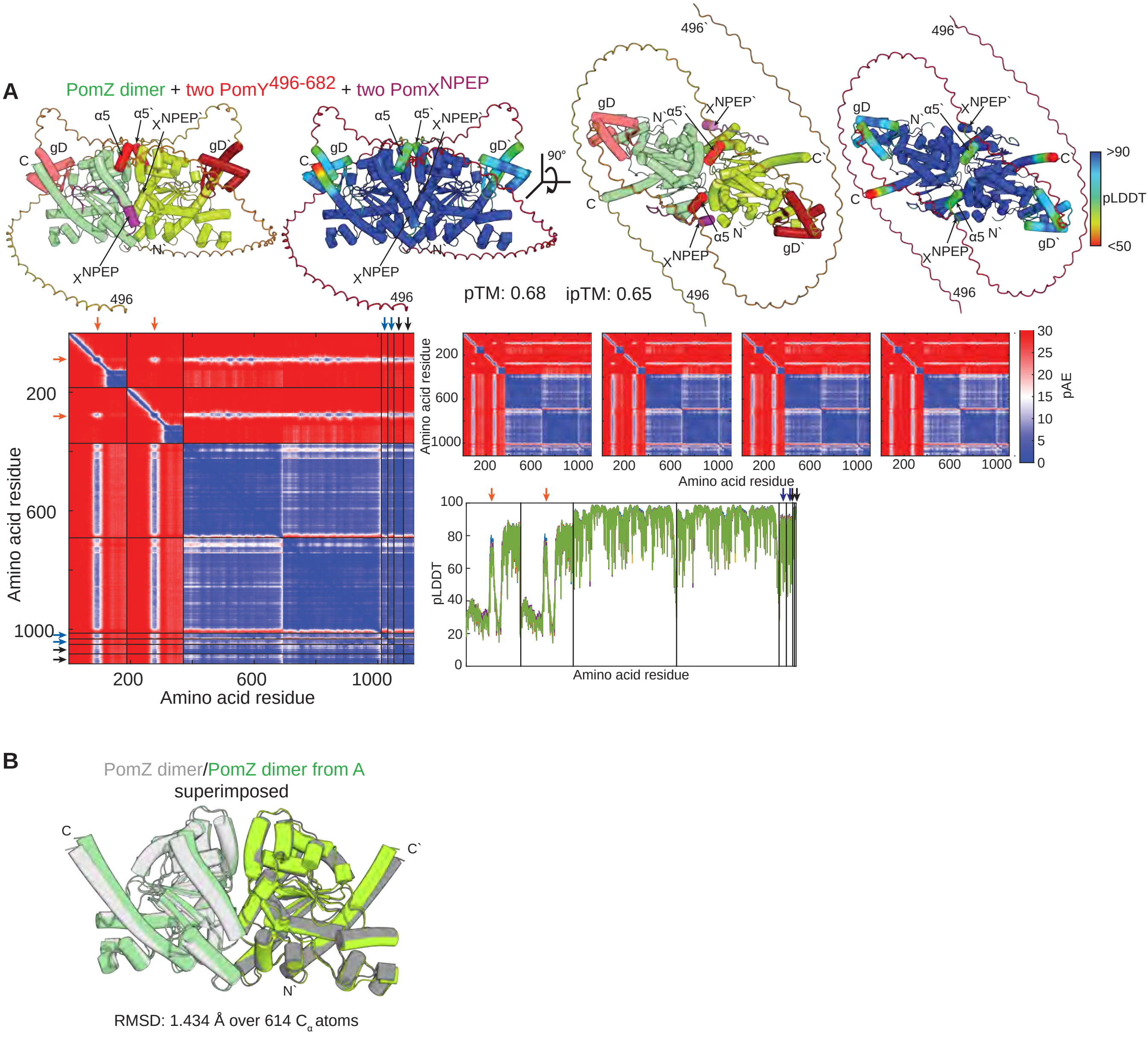
A
PomZ dimer + two PomY496-682 + two PomXNPEP
α5
α5`
XNPEP`
gD
gD`
C
C`
N`
XNPEP
496
496`
90°
pTM: 0.68 ipTM: 0.65
>90
pLDDT
<50
Amino acid residue
pAE
pLDDT
B
PomZ dimer/PomZ dimer from A
superimposed
RMSD: 1.434 Å over 614 Cα atoms

Figure S13. AlphaFold3-based structural model of the PomZ/PomY$^{496-682}$/PomX^{NPEP} interactions

(A) Top panels, model rank 1 of PomZ dimer with two molecules of PomY$^{496-682}$ and two molecules of PomX^{NPEP}. In the left panels, the model is shown with the PomZ dimer in shades of green, the IDRs in PomY$^{496-682}$ in brown, α5 in red, the gD domain in dark red, and PomX^{NPEP} in purple. In the right panels, the model is colored according to pLDDT score. PomZ was modeled with ATP and Mg^{2+} but the ATP molecules and Mg^{2+} are not included for simplicity. Lower panels, pLDDT plot and pAE plots for the five models generated. The black lines separate the two PomZ protomers, the two PomY$^{496-682}$ molecules and the two PomX^{NPEP} molecules; black arrows, ATP and Mg^{2+}, red arrows α5 of PomY$^{496-682}$, and purple arrows PomX^{NPEP}. Model rank 1 is shown in high magnification and was selected for further analyses. Note that the model is of low confidence based on pTM and ipTM scores. The large proportion of IDRs in PomY$^{496-682}$, which have low pLDDT scores, causes these low values.

B) Structural alignment of the PomZ dimer alone with the PomZ dimer complexed with PomY$^{496-682}$/PomX^{NPEP}. The PomZ dimer alone is in shades of grey and the PomZ dimer complexed with PomY$^{496-682}$/PomX^{NPEP} is in shades of green.

A

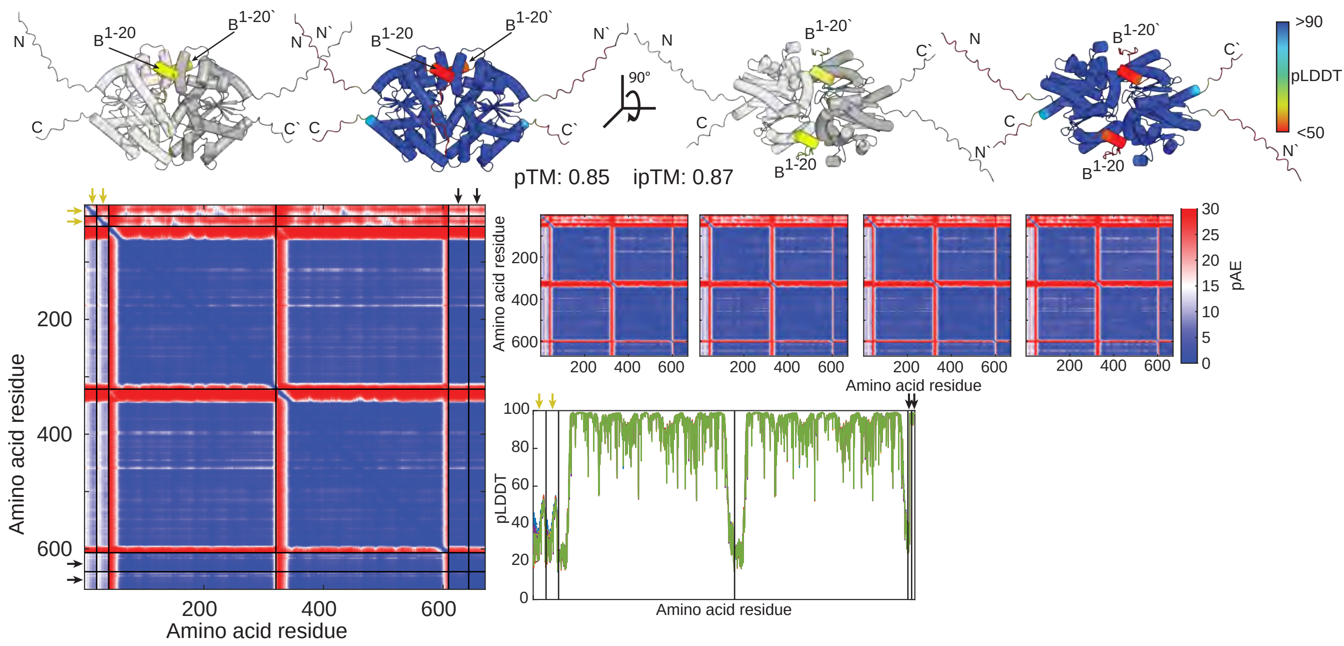

ParA dimer + two ParB1-20
N
B1-20
B1-20`
N`
C
C`
90°
pLDDT
>90
<50
pTM: 0.85 ipTM: 0.87
Amino acid residue
200
400
600
pAE
30
25
20
15
10
5
0
pLDDT
100
80
60
40
20
0

B

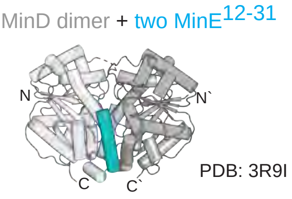

MinD dimer + two MinE12-31
N
N`
C
C`
PDB: 3R9I

Figure S14. AlphaFold3-based structural model of the ParA/$ParB^{1-20}$ interaction

(A) Top panels, model rank 1 of ParA dimer with two molecules $ParB^{1-20}$. In the left panels, the model is shown with the ParA dimer in shades of grey, and the two $ParB^{1-20}$ molecules in yellow. In the right panels, the model is colored according to pLDDT score. ParA was modeled with ATP and Mg^{2+} but the ATP molecules and Mg^{2+} are not included for simplicity. Lower panels, pLDDT plot and pAE plots for the five generated models. The black lines separate the two ParA protomers and the two $ParB^{1-20}$ molecules; black arrows, ATP and Mg^{2+}, and yellow arrows $ParB^{1-20}$. Model rank 1 is shown in high magnification and was selected for further analyses. The ParA and ParB proteins analyzed are those of *M. xanthus* and are involved in chromosome segregation (8).

(B) Solved structure of the *E. coli* MinD dimer (grey shades) with two molecules of $MinE^{12-31}$ (cyan) (9).

Supplementary Table 1. *M. xanthus* strains used in this work.

Strain	Relevant charateristics [1]	Reference
SA4420	Δ*mglA*	(10)
SA4779	Δ*mglA* Δ*pomY*	(1)
SA7064	Δ*mglA* Δ*pomY*/*attB*::P_{nat} *pomY-mCh* (pDS8)	(1)
SA11833	Δ*mglA* Δ*pomY*/*attB*::P_{nat} *pomY*$^{\Delta 496\text{-}682}$-*mCh* (pPK82)	This study
SA3108	Δ*pomZ*	(11)
SA4772	Δ*mglA* Δ*pomZ*	This study
SA4798	Δ*mglA* Δ*pomZ*/*attB*::P_{nat} *pomZ-mCh* (pKA28)	This study
SA11827	Δ*mglA* Δ*pomZ* *pomY*$^{\Delta mIDR}$	This study
SA11828	Δ*mglA* Δ*pomZ* *pomY*$^{\Delta mIDR}$/*attB*::P_{nat} *pomZ-mCh* (pKA28)	This study

[1] P_{nat} indicates that genes were expressed from their native promoter.

[2] Plasmids integrated in a single copy at the Mx8 *attB* site are listed in brackets.

Supplementary Table 2. Plasmids used in this work

Plasmid	Relevant characteristics [1]	Reference
pET24b+	Expression vector for His_6-tagged proteins	Novagen
pTB146	P_{T7}-His_6-Sumo; Amp^R	(12)
pBJ114	Km^R; *galK*$^+$; vector for generating in-frame deletions; Km^R	(13)
pSL16	Δ*mglA*; *galK*$^+$; Km^R (for in-frame deletion of *mglA*)	(10)
pSWU30	*attP*; Tet^R	(14)
pSW105	P_{pilA}; *attP*; Km^R	(15)
pMR3690	Vector for pAH204	(16)
pKNT25	Vector for pDS52 and pDS64	(17)
pKA3	Overexpression of His_6-PomZ; Amp^R	(11)
pDS3	Overexpression of PomY-His_6; Km^R	(2)
pAH194	Overexpression of PomY-mCh-His_6; Km^R	(1)
pAH196	Overexpression of $PomY^{CC}$-mCh-His_6; Km^R	(1)
pAH197	Overexpression of $PomY^{HEAT}$-mCh-His_6; Km^R	(1)
pAH198	Overexpression of $PomY^{mIDR}$-mCh-His_6; Km^R	(1)
pAH187	Overexpression of $PomY^{CC\_HEAT}$-mCh-His_6; Km^R	(1)
pAH195	Overexpression of $PomY^{HEAT\_mIDR}$-mCh-His_6; Km^R	(1)
pAH217	Overexpression of $PomY^{CC\_mIDR}$-mCh-His_6; Km^R	This study
pPK57	Overexpression of $PomY^{\Delta 438-488}$-mCh-His_6; Km^R	This study
pPK67	Overexpression of $PomY^{\Delta 496-623}$-mCh-His_6; Km^R	This study
pPK68	Overexpression of $PomY^{\Delta 624-682}$-mCh-His_6; Km^R	This study
pPK83	Overexpression of His_6-Sumo-$PomY^{mIDR}$-mCh; Amp^R	This study
pPK84	Overexpression of His_6-Sumo-$PomY^{496-623}$-mCh; Amp^R	This study

pPK85	Overexpression of His_6-Sumo-PomY$^{624-682}$-mCh; Amp^R	This study
pPK86	Overexpression of His_6-Sumo-PomY$^{496-682}$-mCh; Amp^R	This study
pPK72	Overexpression of PomY$^{624-682}$-mCh-His_6; Km^R	This study
pGBT9	Y2H plasmid expressing Gal4BD	Clontech
pDS82	Y2H plasmid expressing Gal4BD-PomY	(2)
pDS139	Y2H plasmid expressing Gal4BD-PomY^{CC_HEAT}	This study
pAH274	Y2H plasmid expressing Gal4BD-PomY^{mIDR}	This study
pGAD424	Y2H plasmid expressing Gal4AD	Clontech
pDS83	Y2H plasmid expressing Gal4AD-PomZ	(2)
pDS52	PomZ^{G62V}; Km^R<br>(template for pPK90)	This study
pDS64	PomZ^{K268E}; Km^R<br>(template for pPK91)	This study
pKA12	His_6-PomZ^{D90A}; Amp^R<br>(template for pPK89)	This study
pPK90	Y2H plasmid expressing Gal4AD-PomZ^{G62V}	This study
pPK91	Y2H plasmid expressing Gal4AD-PomZ^{K268E}	This study
pPK89	Y2H plasmid expressing Gal4AD-PomZ^{D90A}	This study
pDS8	P_{nat} PomY-mCh; *attP*; Tet^R	(2)
pPK82	P_{nat} PomY$^{\Delta 496-682}$-mCh; *attP*; Tet^R	This study
pPK78	PomY$^{\Delta mIDR}$; *galK*$^+$; Km^R<br>(for in-frame deletion of mIDR)	This study
pKA28	P_{nat} PomZ-mCh; *attP*; Tet^R	(11)
pDS7	P_{pilA}-PomY-mCh, Km^R<br>(template for pPK35 & pPK53)	(2)
pAH204	P_{pilA} PomY^{CC_mIDR}-mCh; *attP*; Km^R<br>(template for pAH217)	This study
pPK53	P_{pilA} PomY$^{\Delta 438-488}$-mCh; *attP*; Km^R<br>(template for pPK57)	This study

pPK61	P_{pilA} PomY^{mIDR}-mCh; *attP*; Km^{R}<br>(template for pPK67)	This study
pPK38	P_{pilA} PomY$^{\Delta 624\text{-}682}$-mCh; *attP*; Km^{R}<br>(template for pPK68)	This study
pPK71	PomY$^{496\text{-}623}$-mCh-His$_{6}$; Km^{R}<br>(template for pPK84)	This study
pPK74	PomY$^{496\text{-}682}$-mCh-His$_{6}$; Km^{R}<br>(template for pPK86)	This study
pPK35	P_{pilA}-PomY$^{CC\text{-}mIDR}$-mCh; *attP*; Km^{R}<br>(template for pAH217)	This study
pAH107	PomZ^{G62V}; Km^{R}<br>(template for pPK90)	This study
pAH110	PomZK268E ; KmR<br>(template for pPK91)	This study
pDS43	PomZ-mCh; Km^{R}<br>(template for pAH107 & pAH110)	(2)

[1] P_{nat} and P_{pilA} indicate the native promoter and the *pilA* promoter, respectively.

Supplementary Table 3. Oligonucleotides used in this work

Oligo-nucleotide	Sequence (5'-3')
DS14	GCGCATATGAGCGACGAGCGTCCG
DS274	GCGAAGCTTCTTGTACAGCTCGTCCATGC
mcherry stop rev	GGGAAGCTTTTACTTGTACAGCTCGTC
PK87	CGATATTATTGAGGCTCACAGAGAACAGATTGGTGGTATGCTGGAGCAGCCTCGTCCGGTGGTGGCTTCGG
PK86	CCTTTCGGGCTTTGTTAGCAGCCGGATCCTCATCACTTGTACAGCTCGTCCATGCCGCCGGTGGAGTGG
PK85	CGATATTATTGAGGCTCACAGAGAACAGATTGGTGGTATGCCGGTGGGGCCTGTGCAGGGGCGAGC
PK88	CGATATTATTGAGGCTCACAGAGAACAGATTGGTGGTATGGCGTCACCGGTGGAATCCCTCTGCTCGCAGATGC
PK85	CGATATTATTGAGGCTCACAGAGAACAGATTGGTGGTATGCCGGTGGGGCCTGTGCAGGGGCGAGCAGGCCCC
AH230	GCGGAATTCCTGGAGCAGCCTCGTCCGG
AH231	GCGAGATCTTCAAGCGGCGAAGTATTTGTG
Mxan0635 fwd SalI linker	GCGGTCGACCTATGGAAGCGCCGACGTACAGC
Mxan0635 rev stop BglII	GCGAGATCTTCAGCCGGCCTGCTGGGTGCC
DS1	CCGGAATTCCGACGAGCAGTTGAGCACCAG
PK84	CCGGCGGCGGATCTGGCGGAACCAGCGCCCTGCGCGAGGC
PK83	GCCTCGCGCAGGGCGCTGGTTCCGCCAGATCCGCCGCCGG
mcherry stop rev HindIII	GGGAAGCTTTTACTTGTACAGCTCGTC
PK64	CGCAAGCTTCGCTGCTCCAGGCCTTCGC
PK65	CGGCTACCTTGCCTCACCGCGCCCGCACAGGC
PK66	GCCTGTGCGGGCGCGGTGAGGCAAGGTAGCCG
PK80	GCGGAATTCCCGCTGCACCTCC
PK77	GCGCATATGGCGTCACCGGTGGAATCCC
Mxan0634 fwd EcoRI	GCGGAATTCGTGAGCGACGAGCGTCCGGAC
Mxan0634 HEAT rev BglII	GCGAGATCTTCACCGCGCCCGCACAGGC
cluster3 prom 1 bioteg	GGGCCACCAAGCCCACCGCC
cluster3 prom 2 bioteg	GGCAAATTCCCAGGAGGAGG
DS40	GCGGAATTCTTACTTGTACAGCTCGTC
DS11	CAGCGCTTCCTGCAGGCGCTGGAGCAGCCTCGTCCG
DS4	GCGTCTAGAGTGAGCGACGAGCGTCCG
PK39	CTGGAGCAGCCTCGTGGCCTCGCGCAGGGC
PK40	GCCCTGCGCGAGGCCACGAGGCTGCTCCAG
PK44	GCGCAGGGCGCTGGTGTGGAATCCCTCTGC

PK45	GCAGAGGGATTCCACACCAGCGCCCTGCGC
PK78	GCGCATATGCCGGTGGGGCCTGTGCAGGG
PK21	CGCAGATCTGGCGGATGACGCACTGGGGCGCGAGGGGGC
KA419	CTCATCGACCTCGCCAGCCAGGGCCAC
KA420	GTGGCCCTGGCTGGCGAGGTCGATGAG
Mxan_0635-4 pomZ fwd	GCGTCTAGAGATGGAAGCGCCGACGTAC
Mxan_0635-5 pomZ rev	GCGGGTACCCGGCCGGCCTGCTGGGTGCC
KA413	TCCTGAACTTCAAGGTTGGCACCGGCAAGAC
KA414	GTCTTGCCGGTGCCAACCTTGAAGTTCAGGA
AH91	ATCCGGCAGTGCACCGAGTTCGCGCAGGCCTCC
AH92	GGAGGCCTGCGCGAACTCGGTGCACTGCCGGAT

Supplementary Table 4. Construction of plasmids

Plasmid	Construction
pAH217	A fragment containing PomY^{CC} was amplified from pDS7, which contains PomY-mCh, with the primers DS4 and DS10. A second fragment containing PomY^{mIDR}-mCh was amplified from pDS7 with the primers DS11 and mCh stop rev HindIII. The two fragments were fused with the primers DS4 and mCh stop rev HindIII in a PCR. The fragment and vector pSW105 were digested with XbaI and HindIII and, after dephosphorylation, used for ligation. Competent NEB Turbo cells were transformed with the ligation reaction, and the obtained plasmid pPK35 was sequenced. Subsequently, a fragment containing PomY$^{CC-mIDR}$-mCh was amplified from pPK35, which contains PomY$^{CC-mIDR}$-mCh, with the Primers DS14 and DS40. The fragment and vector pMR3690 were digested with NdeI and EcoRI and, after dephosphorylation, used for ligation. Competent NEB Turbo cells were transformed with the ligation reaction, and the obtained plasmid pAH204 was sequenced. Subsequently, the fragment containing PomY$^{CC-mIDR}$-mCh was amplified from pAH204 with the primers DS14 and DS274. The fragment and vector pET24b+ were digested with NdeI and HindIII and, after dephosphorylation, used for ligation. Competent NEB Turbo cells were transformed with the ligation reaction, and the obtained plasmid pAH217 was sequenced.
pPK57	A fragment containing PomY$^{1-437}$ was amplified from pDS7, which contains PomY-mCh, with the primers DS4 and PK40. A second fragment containing PomY$^{489-682}$-mCh was amplified from pDS7 with the primers PK39 and mCh stop rev HindIII. The two fragments were fused with the primers DS4 and mCh stop rev HindIII in a PCR. The fragment and vector pSW105 were digested with XbaI and HindIII and, after dephosphorylation, used for ligation. Competent NEB Turbo cells were transformed with the ligation reaction, and the obtained plasmid pPK53 was sequenced. Subsequently, the fragment containing PomY$^{\Delta 438-488}$-mCh was amplified from pPK53 with the primers DS14 and mCh stop rev. The fragment and vector pET24b+ were digested with NdeI and HindIII and, after dephosphorylation, used for ligation. Competent NEB Turbo cells were transformed with the ligation reaction, and the obtained plasmid pPK57 was sequenced.
pPK67	A fragment containing PomY$^{1-495}$ was amplified from pDS8, which contains PomY-mCh, with the primers DS4 and PK45. A second fragment containing PomY$^{624-682}$-mCh was amplified from pD8 with the primers PK44 and mCh stop rev HindIII. The two fragments were fused with the primers DS4 and mCh stop rev HindIII in a PCR. The fragment and vector pSW105 were digested with XbaI and HindIII and, after dephosphorylation, used for ligation. Competent NEB Turbo cells were transformed with the ligation reaction, and the obtained plasmid pPK61 was sequenced. Subsequently, the fragment containing PomY$^{\Delta 496-623}$-mCh was amplified from pPK61 with the primers DS14 and DS274. The fragment and vector pET24b+ were digested with NdeI and HindIII and, after dephosphorylation, used for ligation. Competent NEB Turbo cells were transformed with the ligation reaction, and the obtained plasmid pPK67 was sequenced.
pPK68	A fragment containing PomY$^{\Delta 624-682}$ was amplified from pDS8, which contains PomY-mCh, with the Primers DS4 and PK21. The fragment was digested with XbaI and BglII, and the vector pSWU30 was digested with XbaI and BamHI and, after dephosphorylation, used for ligation. Competent NEB Turbo cells were transformed with the ligation reaction, and the obtained plasmid pPK38 was sequenced. Subsequently, the fragment containing PomY$^{\Delta 624-682}$-mCh was amplified from pPK38 with the primers DS14 and DS274. The fragment and vector pET24b+ were digested with NdeI and HindIII and, after dephosphorylation, used for ligation. Competent NEB Turbo cells were transformed with the ligation reaction, and the obtained plasmid pPK68 was sequenced.
pPK83	A fragment containing PomY^{mIDR}-mCh was amplified from pAH198, which contains PomY^{mIDR}-mCh, with the Primers PK87 and PK86. The Fragment was

	inserted into the vector pTB146 with the Gibson Assembly Cloning Kit, NEB #E5510S.
pPK84	A fragment containing PomY$^{496-623}$-mCh was amplified from pPK68, which contains PomY$^{\Delta 624-682}$-mCh, with the Primers PK78 and DS274. The fragment and vector pET24b+ were digested with NdeI and HindIII and, after dephosphorylation, used for ligation. Competent NEB Turbo cells were transformed with the ligation reaction, and the obtained plasmid pPK71 was sequenced. Subsequently, the fragment containing PomY$^{496-682}$-mCh was amplified from pPK71 with the primers PK85 and PK86. The Fragment was inserted into the vector pTB146 with the Gibson Assembly Cloning Kit, NEB #E5510S.
pPK85	A fragment containing PomY$^{624-682}$-mCh was amplified from pAH194, which contains His_6-PomY-mCh, with the Primers PK77 and DS274. The fragment and vector pET24b+ were digested with NdeI and HindIII and, after dephosphorylation, used for ligation. Competent NEB Turbo cells were transformed with the ligation reaction, and the obtained plasmid pPK72 was sequenced. Subsequently, the fragment containing PomY$^{624-682}$-mCh was amplified from pPK72 with the primers PK88 and PK86. The Fragment was inserted into the vector pTB146 with the Gibson Assembly Cloning Kit, NEB #E5510S.
pPK86	A fragment containing PomY$^{496-682}$-mCh was amplified from pAH194, which contains His_6-PomY-mCh, with the Primers PK78 and DS274. The fragment and vector pET24b+ were digested with NdeI and HindIII and, after dephosphorylation, used for ligation. Competent NEB Turbo cells were transformed with the ligation reaction, and the obtained plasmid pPK74 was sequenced. Subsequently, the fragment containing PomY$^{496-682}$-mCh was amplified from pPK74 with the primers PK85 and PK86. The Fragment was inserted into the vector pTB146 with the Gibson Assembly Cloning Kit, NEB #E5510S.
pDS139	A fragment containing PomY$^{CC-HEAT}$ was amplified from DK1622 genomic DNA with the Primers Mxan 0634 fwd EcoRI and Mxan0634 HEAT rev BglII. The fragment was digested with EcoRI and BglII, and the vector pGBT9 was digested with XbaI and BamHI and, after dephosphorylation, used for ligation. Competent NEB Turbo cells were transformed with the ligation reaction, and the obtained plasmid pDS139 was sequenced.
pAH274	A fragment containing PomY^{mIDR} was amplified from DK1622 genomic DNA with the Primers AH230 and AH231. The fragment and vector pGBT9 were digested with EcoRI and BamHI and, after dephosphorylation, used for ligation. Competent NEB Turbo cells were transformed with the ligation reaction, and the obtained plasmid pAH274 was sequenced.
pPK90	The G62V point mutation in PomZ was inserted by amplifying the plasmid pDS43 with the primers KA413 and KA414, carrying the point mutation, with the QuikChange II XL Site-Directed Mutagenesis Kit (Agilent), and the obtained plasmid pAH107 was sequenced. Subsequently, a fragment containing PomZ^{G62V} was amplified from pAH107, which contains PomZ^{G62V}, with the Primers Mxan_0635-4 pomZ fwd and Mxan_0635-5 pomZ rev. The fragment and vector pKNT25 were digested with XbaI and KpnI and, after dephosphorylation, used for ligation. Competent NEB Turbo cells were transformed with the ligation reaction, and the obtained plasmid pDS52 was sequenced. Subsequently, the fragment containing PomZ^{G62V} was amplified from pDS52 with the primers Mxan 0634 fwd SalI linker and Mxan0635 rev stop BglII. The fragment and vector pGAD424 were digested with SalI and BglII and, after dephosphorylation, used for ligation. Competent NEB Turbo cells were transformed with the ligation reaction, and the obtained plasmid pPK90 was sequenced.
pPK91	The K268E point mutation in PomZ was inserted by amplifying the whole plasmid pDS43 with the primers AH91 and AH92, carrying the point mutation, with the QuikChange II XL Site-Directed Mutagenesis Kit (Agilent), and the obtained plasmid pAH110 was sequenced. Subsequently, a fragment containing PomZ^{K268E} was amplified from pAH110, which contains PomZ^{K268E}, with the

	Primers Mxan_0635-4 pomZ fwd and Mxan_0635-5 pomZ rev. The fragment and vector pKNT25 were digested with XbaI and KpnI and, after dephosphorylation, used for ligation. Competent NEB Turbo cells were transformed with the ligation reaction, and the obtained plasmid pDS64 was sequenced. Subsequently, the fragment containing $PomZ^{K268E}$ was amplified from pDS64 with the primers Mxan 0634 fwd SalI linker and Mxan0635 rev stop BglII. The fragment and vector pGAD424 were digested with SalI and BglII and, after dephosphorylation, used for ligation. Competent NEB Turbo cells were transformed with the ligation reaction, and the obtained plasmid pPK91 was sequenced.
pPK89	The D90A point mutation in PomZ was inserted by amplifying the whole plasmid pKA28 with the primers KA419 and KA420, carrying the point mutation, with the QuikChange II XL Site-Directed Mutagenesis Kit (Agilent), and the obtained plasmid pKA12 was sequenced. Subsequently, the fragment containing $PomZ^{D90A}$ was amplified from pDS64 with the primers Mxan 0634 fwd SalI linker and Mxan0635 rev stop BglII. The fragment and vector pGAD424 were digested with SalI and BglII and, after dephosphorylation, used for ligation. Competent NEB Turbo cells were transformed with the ligation reaction, and the obtained plasmid pPK89 was sequenced.
pPK82	A fragment containing $PomY^{1-495}$ was amplified from pDS8, which contains PomY-mCh, with the primers DS1 and PK84. A second fragment containing mCh was amplified from pD8 with the primers PK83 and mCh stop rev HindIII. The two fragments were fused with the primers DS1 and mCh stop rev HindIII in a PCR. The fragment and vector pSWU30 were digested with EcoRI and HindIII and, after dephosphorylation, used for ligation. Competent NEB Turbo cells were transformed with the ligation reaction, and the obtained plasmid pPK82 was sequenced.
pPK78	A fragment containing 500bp upstream of PomYmIDR was amplified from pAH194, which contains PomY-mCh, with the primers PK64 and PK65. A second fragment containing 500bp of the genomic region immediately following the *pomY* locus, was amplified from genomic SA4772 DNA with the primers PK66 and PK80. The two fragments were fused with the primers PK64 and PK80 in a PCR. The fragment and vector pBJ114 were digested with EcoRI and HindIII and, after dephosphorylation, used for ligation. Competent NEB Turbo cells were transformed with the ligation reaction, and the obtained plasmid pPK78 was sequenced.
pPK72	A fragment containing $PomY^{624-682}$-mCh was amplified from pAH194, which contains PomY-mCh-His_6, with the Primers PK77 and DS274. The fragment and vector pET24b+ were digested with NdeI and HindIII and, after dephosphorylation, used for ligation. Competent NEB Turbo cells were transformed with the ligation reaction, and the obtained plasmid pPK72 was sequenced.

Supplementary References

1. Ramm B, Schumacher D, Harms A, Heermann T, Klos P, Muller F, Schwille P, Søgaard-Andersen L. 2023. Biomolecular condensate drives polymerization and bundling of the bacterial tubulin FtsZ to regulate cell division. Nat Commun 14:3825.

2. Schumacher D, Bergeler S, Harms A, Vonck J, Huneke-Vogt S, Frey E, Søgaard-Andersen L. 2017. The PomXYZ Proteins Self-Organize on the Bacterial Nucleoid to Stimulate Cell Division. Dev Cell 41:299-314.

3. Kahmann JD, Wecking DA, Putter V, Lowenhaupt K, Kim YG, Schmieder P, Oschkinat H, Rich A, Schade M. 2004. The solution structure of the N-terminal domain of E3L shows a tyrosine conformation that may explain its reduced affinity to Z-DNA *in vitro*. Proc Natl Acad Sci U S A 101:2712-7.

4. Schwartz T, Rould MA, Lowenhaupt K, Herbert A, Rich A. 1999. Crystal structure of the Zalpha domain of the human editing enzyme ADAR1 bound to left-handed Z-DNA. Science 284:1841-5.

5. de Rosa M, Zacarias S, Athanasiadis A. 2013. Structural basis for Z-DNA binding and stabilization by the zebrafish Z-DNA dependent protein kinase PKZ. Nucleic Acids Res 41:9924-33.

6. Ha SC, Kim D, Hwang HY, Rich A, Kim YG, Kim KK. 2008. The crystal structure of the second Z-DNA binding domain of human DAI (ZBP1) in complex with Z-DNA reveals an unusual binding mode to Z-DNA. Proc Natl Acad Sci U S A 105:20671-6.

7. Chu CH, Yen CY, Chen BW, Lin MG, Wang LH, Tang KZ, Hsiao CD, Sun YJ. 2019. Crystal structures of HpSoj-DNA complexes and the nucleoid-adaptor complex formation in chromosome segregation. Nucleic Acids Res 47:2113-2129.

8. Harms A, Treuner-Lange A, Schumacher D, Søgaard-Andersen L. 2013. Tracking of chromosome and replisome dynamics in *Myxococcus xanthus* reveals a novel chromosome arrangement. PLoS Genet 9:e1003802.

9. Park K-T, Wu W, Battaile Kevin P, Lovell S, Holyoak T, Lutkenhaus J. 2011. The Min oscillator uses MinD-dependent conformational changes in MinE to spatially regulate cytokinesis. Cell 146:396-407.

10. Miertzschke M, Körner C, Vetter IR, Keilberg D, Hot E, Leonardy S, Søgaard-Andersen L, Wittinghofer A. 2011. Structural analysis of the Ras-like G protein MglA and its cognate GAP MglB and implications for bacterial polarity. EMBO J 30:4185-97.

11. Treuner-Lange A, Aguiluz K, van der Does C, Gomez-Santos N, Harms A, Schumacher D, Lenz P, Hoppert M, Kahnt J, Munoz-Dorado J, Søgaard-Andersen L. 2013. PomZ, a ParA-like protein, regulates Z-ring formation and cell division in *Myxococcus xanthus*. Mol Microbiol 87:235-53.

12. Bendezu FO, Hale CA, Bernhardt TG, de Boer PA. 2009. RodZ (YfgA) is required for proper assembly of the MreB actin cytoskeleton and cell shape in *E. coli*. EMBO J 28:193-204.

13. Julien B, Kaiser AD, Garza A. 2000. Spatial control of cell differentiation in *Myxococcus xanthus*. Proc Natl Acad Sci U S A 97:9098-103.

14. Wu SS, Kaiser D. 1997. Regulation of expression of the *pilA* gene in *Myxococcus xanthus*. J Bacteriol 179:7748-58.

15. Jakovljevic V, Leonardy S, Hoppert M, Søgaard-Andersen L. 2008. PilB and PilT are ATPases acting antagonistically in type IV pilus function in *Myxococcus xanthus*. J Bacteriol 190:2411-21.

16. Iniesta AA, Garcia-Heras F, Abellon-Ruiz J, Gallego-Garcia A, Elias-Arnanz M. 2012. Two systems for conditional gene expression in *Myxococcus xanthus* inducible by isopropyl-beta-D-thiogalactopyranoside or vanillate. J Bacteriol 194:5875-85.

17. Karimova G, Pidoux J, Ullmann A, Ladant D. 1998. A bacterial two-hybrid system based on a reconstituted signal transduction pathway. Proc Natl Acad Sci U S A 95:5752-6.
